# LncRNA SAMD12-AS1 Suppresses Proliferation and Migration of Hepatocellular Carcinoma via p53 Signaling Pathway

**DOI:** 10.1155/2022/9096365

**Published:** 2022-08-23

**Authors:** Juan Wang, Yuan Zhou, Chunyan Gu, Fang Ming, Ying Zhang

**Affiliations:** ^1^Department of Infectious Disease, Nantong Third Affiliated Hospital of Nantong University, Nantong, China; ^2^Department of Hepatobiliary Surgery, Tumor Hospital Affiliated to Nantong University, Nantong Tumor Hospital, Nantong, China; ^3^Department of Pathology, Nantong Third Affiliated Hospital of Nantong University, Nantong, China

## Abstract

**Purpose:**

Assessment of lncRNA SAMD12-AS1 expression in liver cancer tissues and cell lines to investigate the underlying molecular mechanisms that regulate liver cancer cell growth, development, invasion, and migration.

**Methods:**

The lncRNA SAMD12-AS1 expression in tumor tissues of 32 liver cancer patients was measured by real-time PCR, and its effect on the clinicopathological manifestations and liver cancer patients' prognosis was determined. LncRNA SAMD12-AS1 overexpression and knockdown in liver cancer cell lines were established by cell transfection. The effects of lncRNA SAMD12-AS1 knockdown and overexpression on liver cancer cell growth, development, invasion, and migration were determined by MTT, Transwell, and clonogenic assays. Furthermore, its effects on the expression of E-cadherin, vimentin, p53, and p21 in hepatocellular carcinoma cells were determined by Western blot assay.

**Results:**

The level of lncRNA SAMD12-AS1 expression in tumor tissues was remarkably higher than that in paracancerous liver tissues (*p* < 0.01). It was found that the lncRNA SAMD12-AS1 expression was largely correlated with TNM stage of tumor, vascular invasion, and hepatitis B surface (HBs) antigen in liver cancer patients (*p* < 0.05). Cell function experiments showed that lncRNA SAMD12-AS1 overexpression promoted liver cancer development, migration, and invasion (*p* < 0.05), while lncRNA SAMD12-AS1 knockdown inhibited the activity of liver cancer cells to invade and migrate (*p* < 0.05). Western blot analysis showed that overexpression of lncRNA SAMD12-AS1 markedly inhibited p21, p53, and E-cadherin expression and promoted vimentin expression. Conversely, knockdown of lncRNA SAMD12-AS1 significantly promoted p21, p53, and E-cadherin expression and inhibited vimentin expression (*p* < 0.05).

**Conclusion:**

LncRNA SAMD12-AS1 is associated with the TNM stage and vascular invasion of liver cancer. It promotes liver cancer cell development, invasion, and migration by regulating p53 expression. Thus, lncRNA SAMD12-AS1 could be a novel biological target for the treatment of liver cancer.

## 1. Introduction

Hepatocellular carcinoma is one of the most prevalent malignant tumors with increasing incidence globally [1]. Although the early diagnosis method of liver cancer has been improved in recent years, due to its insidious onset and lack of obvious symptoms in the early stage, most of the diagnoses were made during the middle and advance stages. This makes it difficult to carry out effective surgical treatment, which is responsible for a relatively low survival rate of 5 years [2, 3]. Studies suggest that the postoperative recurrence rate in liver cancer patients is high (70%), whereas the 5-year survival rate is <50%. Recurring and metastasized tumors are the core reasons for treatment failure against liver cancer. To improve the therapeutic effect of liver cancer, it is important to determine the molecular mechanisms that affect liver cancer cell migration and invasion [4]. Hence, an in-depth study related to the molecular mechanism involved in liver cancer prevalence and progression is greatly important for the discovery of novel biomarkers for diagnosis, treatment, and improving the prognosis of liver cancer patients.

Long noncoding RNAs (lncRNAs) are RNAs with more than 200 nucleotides (nucleotide, nt) and either lack or have only weak protein-coding capacity [5]. LncRNAs are seen to regulate the expression of genes at epigenetic, transcriptional, post-transcriptional, translational, and post-translational levels in multiple ways and are readily involved in the modulation of tumor progression, metastasis, and recurrence [6]. Previously, it was confirmed that lncRNAs play a role in the proliferation, apoptosis, invasion, and metastasis; angiogenesis and prognosis of liver cancer cells; and various biological processes that are associated with liver cancer progression [7]. For example, a low level of lncRNA ZNF385D-AS2 expression observed in liver cancer was highly correlated with the patient's TMN stage. Cox analysis revealed that low expression of lncRNA ZNF385D-AS2 was a prognostic variable that was independent of overall survival in liver cancer patients [8]. One of the oncogenic lncRNAs is lncRNA XIST, which promotes liver cancer growth, invasion, development, and metastasis via miR-200b-3p/ZEB1/2 signaling axis [9]. The lncRNA MALAT1 regulates the stem cell properties of liver cancer through sponging miR-375 and regulates YAP1 expression, thereby regulating the promotion of liver cancer recurrence and metastasis [10]. In addition, lncRNAs can participate in HBV virus replication, hence modulating liver cancer incidence and development. For example, the lncRNA PCNAP1 enhances HBV replication by controlling the signaling of cccDNA (miR-154/HBV/PCNA) and PCNAP1/PCNA signaling stimulating hepatocarcinogenesis [11]. HBx of HBV can significantly downregulate the expression of LINC01010 in hepatocytes, and LINC01010 can decrease the cellular levels of insoluble vimentin. The LINC01010 interferes with vimentin polymerization and then inhibits the proliferation and migration ability of liver cancer cells. Thus, LINC01010 is a potential tumor suppressor that can inhibit HBV-related liver cancer development [12]. The lncRNA SAMD12-AS1 is a new cancer-promoting lncRNA, and its function is less studied. For example, studies have found that lncRNA SAMD12-AS1 expression in tumor tissue of glioma patient samples was greatly increased compared with paracancerous tissues, and higher lncRNA SAMD1-AS1 expression was associated with a higher incidence of lymph node or distant metastasis. Knockout of lncRNA SAMD12-AS1 largely suppressed glioma cell proliferation, and their invasive and migratory abilities [13]. The lncRNA SAMD12-AS1 was seen to be significantly expressed in cancer tissues and cell lines of gastric cancer. Previous studies have indicated that the lncRNA SAMD12-AS1 may have a biological role in gastric cancer by direct interaction with DNMT1 and promotes DNMT1 to inhibit the p53 signaling pathway [14]. In addition, the lncRNA SAMD12-AS1 is related to the progression of hepatocellular carcinoma and inhibition of apoptosis by interacting with NPM1 [15]. Despite multiple studies on the molecular mechanism and function of lncRNA SAMD12-AS1 in other cancers, its function in hepatocellular carcinoma remains unknown. This study aims to explore the biological function and expression of lncRNA SAMD12-AS1 in liver cancer tissues and provide targets for prognosis and therapy of liver cancer.

## 2. Material and Method

### 2.1. Tissue Sample Collection

This study was approved by our hospital's ethical committee, and the voluntarily signed informed consent forms were obtained from all the patients or their families. Totally, 32 patients with liver cancer were selected for tissue sample collection. These patients were treated in the general surgery department of our hospital. The paracancerous liver tissues were collected at a distance ≥3 cm beyond tumor edge, and pathological examination showed the presence of no cancer cells. All patients were pathologically diagnosed and the follow-up data were complete, and the patients did not receive antitumor therapy before surgery. The postoperative pathological diagnosis is hepatocellular carcinoma. Exclusion criteria: (1) patients with other tumors; (2) preoperative chemotherapy, radiotherapy, and other treatments; and (3) patients with liver, heart, kidney, and other major organ dysfunction. The specimens were taken out of the body and stored in cryovials followed by liquid nitrogen snap-freezing, prior to −80°C storage in an ultra-low-temperature freezer.

### 2.2. Cell Culture and Transfection

Hepatocellular carcinoma cell lines (Huh7, HCCLM3, SMMC-7721, and HepG2) and human immortalized hepatocytes LO2 were cultured at a constant temperature of 37°C in a cell incubator with 5% CO_2_. The cell culture medium was refreshed every 2 days, and the cells were passaged every 4–5 days. When the growth reached about 80%, they were passaged by 0.25% trypsin digestion. The LO2 cells were routinely cultured in the complete medium of DMEM (Gibco) with 10% FBS (Gibco).

The hepatocellular carcinoma cells at logarithmic growth phase were seeded in 6-well plates (2 × 105 cells/well). The cells were divided into pcDNA-control group, pcDNA-SAMD12-AS1 group, shRNA-control group, and shSAMD12-AS1 group, three replicates in each group. All the groups were transfected when the cells had grown to 70–80% confluence. The constructed lentiviral vector was incubated with cells, gently mixed for 5 min at room temperature, before being placed at 37°C in a constant temperature cell incubator containing 5% CO_2_ for 6 hours, and replaced with a complete cell culture medium for 48 hours. Later, the transfection efficiency was evaluated and subsequent experimental analysis was performed.

### 2.3. Cell Viability Detection Using CCK-8 Assay

The cells (2 × 10^3^ per well) for the pcDNA-control group, pcDNA-SAMD12-AS1 group, shRNA-control group, and shSAMD12-AS1 group were grown in 96-well plates. Each group was cultured for 24 h, 48 h, 72 h, and 96 h with 3 sets of replicate wells. After culture, the CCK-8 reagent (10 *μ*L) was added and the cells were incubated for another 2 h. Optical density (OD) was measured at 490 nm in the microplate reader. 3 replicates of the experiment were performed, and the mean value was calculated.

### 2.4. Real-Time PCR

Tissues or cells were processed for the extraction of total RNA, following the method provided by the TRIzol kit (Invitrogen, USA). cDNA synthesis was catalyzed by using the PrimeScript™ RT Master Mix kit (Promega, USA). Then, lncRNA ATPT1-AS1 expression was measured by using the ABI PRISM 7700 system using the SYBR-Green PCR Master Mix kit. Reaction parameter settings: denaturation for 60 secs at 95°C, 45 cycles (94°C for 30 secs and 62°C for 45 secs). The relative expression of lncRNA TPT1-AS1 was analyzed by using the 2−△△CT method.

### 2.5. Clonogenic Assay

The transfected pcDNA-control group, pcDNA-SAMD12-AS1 group, shRNA-control group, and shSAMD12-AS1 group were added to 6-well plates with about 600 cells per well. The medium was refreshed after every three days for 14 days. After 4% paraformaldehyde (1 mL) fixation for 30 min, the cells were washed with PBS 3 times, following crystal violet staining (0.1%, 1 mL) for 10 min, and finally PBS washing for 3 times. The number of colonies was counted, and calculation was repeated 3 times.

### 2.6. Transwell Experiment

The artificial basement membrane (Matrigel; Corning, New York, USA) was thawed in advance and then diluted in DMEM medium (serum-free) at a 1 : 8 ratio. After the Transwell chamber (8 *μ*m pore size; Corning, New York, USA) was evenly overlaid with Matrigel on the microporous membrane, the Matrigel was then kept for incubation for 2 h at 37°C. The cells of each group were converted to cell suspension of 1 × 10^4^ cells/mL. 100 *μ*L of each cell suspension was picked and added to the upper chamber of the Transwell chamber. In the lower chamber, DMEM (700 *μ*L) was added. The cells were then allowed to culture for 48 h in an incubator. After incubation, cells present in the upper layer of the microporous membrane were gently wiped out using a cotton swab and then washed 2 times with PBS. For cells in the lower layer of the microporous membrane, 95% ethanol was used for fixation. The cells were then stained with a 0.5% crystal violet staining solution (Beyotime Biotech, China), washed with PBS, viewed, and counted under an inverted microscope (average of 6 fields of view for each group).

### 2.7. Determination of Protein Content by Western Blot

Western blot analysis was performed with the following steps. ①Protein extraction: the cells were disrupted with an ultrasonic tissue disrupter (disruption conditions were 4 times/(tube·300 W)). The disrupted cells were placed on ice at 4°C for 30 min, centrifuged at 12,000 × *g* for 30 min, and the supernatant was discarded. ②Protein quantification: the BCA method was applied for protein quantification (Pierce, Rockford, IL, USA). ③Gel electrophoresis: take 15 *μ*L of the sample to be tested for loading, and use SDS-polyacrylamide gel for electrophoresis analysis until the target molecular weight appears. ④Wet membrane transfer: the protein strips were electrically transferred to Immun-Blot PVDF membrane (Millipore Corp, Atlanta, GA, USA) by wet method. ⑤ Antibody detection: after that, 50 g/L nonfat milk powder was utilized for blocking with constant shaking for 3 h at 20°C. The following antibodies (mouse) were added: anti-human p53 (Abcam), p21(Abcam), E-cadherin (CST), and vimentin (CST). The membrane was kept at 4°C for overnight incubation. The membranes were incubated with horseradish peroxidase-labeled IgG (1 : 1 000) at 37°C for 2 h. Finally, luminescence development and image analysis were carried out.

### 2.8. Statistical Methods

Statistical software (SPSS version 20.0) was applied. Normal distributed data were depicted as mean ± standard deviation (*x* ± *s*). ANOVA was used for intergroup comparison. Measurement data were expressed by rate, and intergroup comparison was analyzed by *χ*^2^ analysis; the test level was *α* = 0.05. Statistically significant difference in value was indicated by *p* < 0.05.

## 3. Result

### 3.1. LncRNA SAMD12-AS1 Overexpressed in Hepatocellular Carcinoma Tissues and Cell Lines

**Figure 1:**
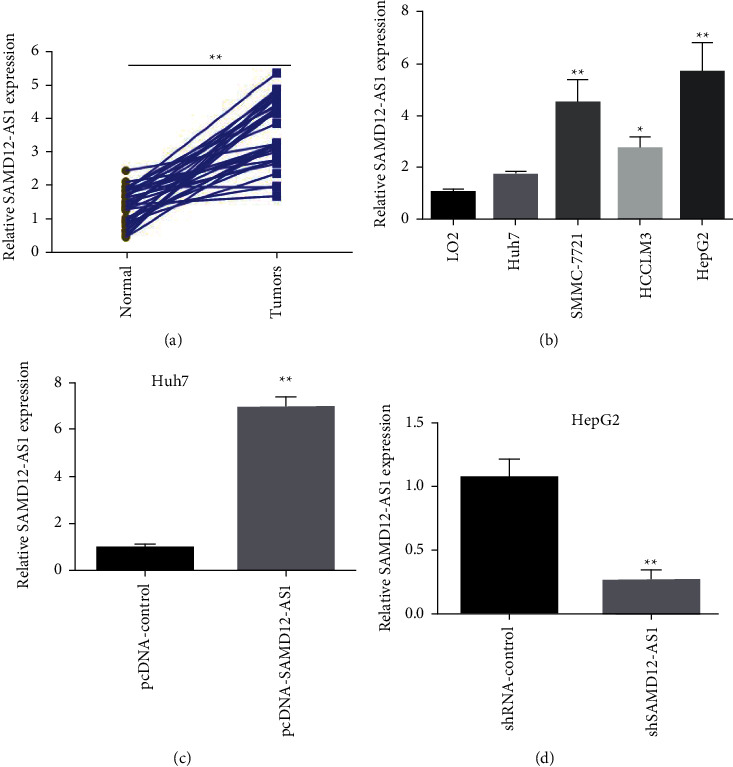
LncRNA SAMD12-AS1 is overexpressed in liver cancerous tissues and cell lines: (a) real-time fluorescence quantitative PCR detection of lncRNA SAMD12-AS1 expression in tumor tissues of 32 liver cancer patients; (b) real-time fluorescence quantitative PCR detection of lncRNA SAMD12-AS1 expression in 4 hepatocellular carcinoma cell lines; (c) lncRNA SAMD12-AS1-overexpressed lentivirus was transfected in Huh7 cells; and (d) lncRNA SAMD12-AS1 knockdown was transfected in HepG2 cells ^∗^*p* < 0.05, ^∗∗^*p* < 0.01.

Real-time fluorescence quantitative PCR data indicated that the expression level of lncRNA SAMD12-AS1 in tumor tissues of 32 liver cancer patients was markedly increased than that in paracancerous liver tissues (*p* < 0.01) (Figure 1(a)). According to the median lncRNA SAMD12-A1S expression in liver cancer tissue, liver cancer patients were grouped into lncRNA SAMD12-AS1 high- and low-expression groups. The lncRNA SAMD12-A1S expression was not associated with tumor size, alpha-fetoprotein (AFP), and degree of differentiation (all *p* > 0.05) but was correlated significantly with tumor TNM stage, vascular invasion, and hepatitis B surface antigen (HBs antigen) (all *p* < 0.05) (Table 1).

Compared with normal hepatocyte LO2, lncRNA SAMD12-AS1 expression in these 4 liver cancer cell lines Huh7, HCCLM3, SMMC-7721, and HepG2 were greatly upregulated, and all the variables showed statistical significance (*p* < 0.05) (Figure 1(b)). Since lncRNA SAMD12-AS1 has the highest expression in HepG2 cells and lowest expression in Huh7 cells among all other liver cancer cell lines in this study, Huh7 and HepG2 cells were selected for overexpression and knockdown experiments, respectively. The results of real-time fluorescence quantitative PCR revealed that in Huh7 cells, in comparison with the pcDNA-control group, transfection of the lentiviral vector (pcDNA-SAMD12-AS1) overexpressing lncRNA SAMD12-AS1 significantly promoted the expression of intracellular lncRNA SAMD12-AS1 (Figure 1(c)), while in HepG2 cells, transfection with a lentiviral vector (shSAMD12-AS1) knocking down lncRNA SAMD12-AS1 greatly reduced the intracellular lncRNA SAMD12-AS1 expression level compared with the shRNA-control cells (Figure 1(d)).

### 3.2. Overexpression or Knockdown of LncRNA SAMD12-AS1 Affects the Proliferation of Hepatocellular Carcinoma Cells

**Figure 2:**
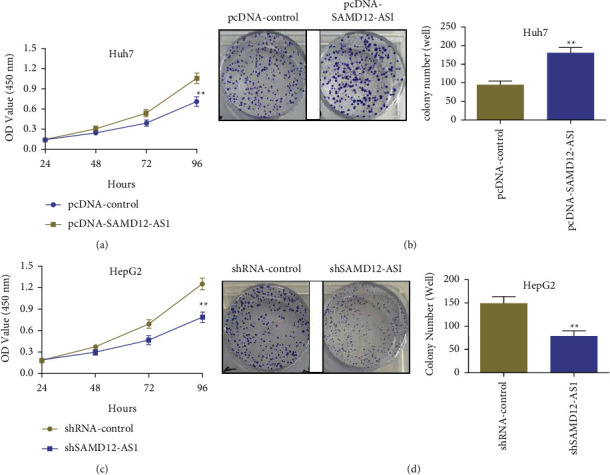
Knockdown or overexpression of lncRNA SAMD12-AS1 can affect liver cancer cell proliferation: (a) CCK-8 assay revealed that lncRNA SAMD12-AS1 overexpression affected the proliferation of liver cancer Huh7 cells; (b) clonogenic assay showing lncRNA SAMD12-AS1 overexpression affected the clonogenic formation of liver cancer Huh7 cells; (c) CCK-8 detection of lncRNA SAMD12-AS1 knockdown affected the proliferative activity of liver cancer HepG2 cells; and (d) clonogenic assay showing lncRNA SAMD12-AS1 knockdown affected the clonogenic activity of hepatocellular carcinoma HepG2 cells. ^∗∗^*p* < 0.01.

CCK-8 assay was performed to evaluate the effect of lncRNA SAMD12-AS1 knockdown and overexpression, on the hepatocellular carcinoma cells. The data disclosed that the overexpression of lncRNA SAMD12-AS1 (pcDNA-SAMD12-AS1) significantly promoted hepatocellular carcinoma cell proliferation in Huh7 cells in comparison with the pcDNA-control group (*p* < 0.01) (Figure 2(a)). The data from the clonogenic assay indicated that the overexpression of lncRNA SAMD12-AS1 (pcDNA-SAMD12 -AS1) promoted colony formation in contrast with the pcDNA-control group (*p* < 0.01) (Figure 2(b)). In HepG2 cells, lncRNA SAMD12-AS1 knockdown (shSAMD12-AS1) largely suppressed hepatocellular carcinoma cell proliferation in comparison with the shRNA-control group (*p* < 0.01) (Figure 2(c)). The clonogenic assay results indicated that knockdown of lncRNA SAMD12-AS1 (shSAMD12-AS1) markedly inhibited colony formation in hepatocellular carcinoma cells compared with the shRNA-control group (*p* < 0.01) (Figure 2(d). The above data indicated that lncRNA SAMD12-AS1 overexpression or knockdown could affect the proliferative activity of hepatocellular carcinoma cells.

### 3.3. Effects of LncRNA SAMD12-AS1 Knockdown or Overexpression on Migration and Invasion of Liver Cancer Cells

**Figure 3:**
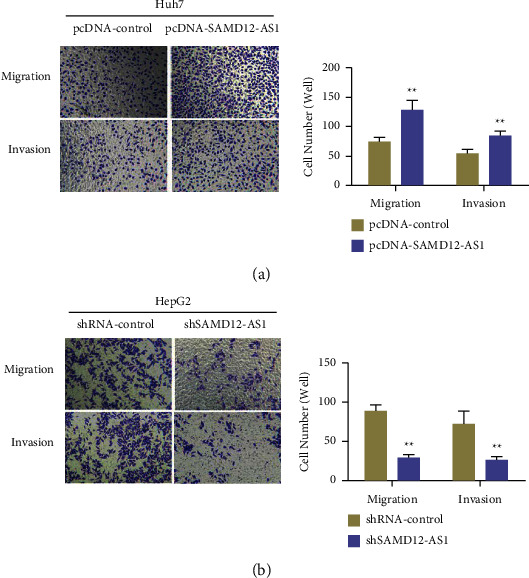
The effect of lncRNA SAMD12-AS1 overexpression and knockdown on the liver cancer cell invasion and migration: (a) lncRNA SAMD12-AS1 overexpression promoted invasion and migration of liver cancer Huh7 cells and (b) knockdown of lncRNA SAMD12-AS1 inhibited the migration and invasion of liver cancer HepG2 cells. ^∗∗^*p* < 0.01.

A number of studies have indicated that metastasis is among the top causes of high mortality of liver cancer. The effect of lncRNA SAMD12-AS1 knockdown or its overexpression on the migratory and invasive capability of liver cancer cells was evaluated by Transwell assay. The results indicated that overexpression of lncRNA SAMD12-AS1 (pcDNA-SAMD12-AS1) significantly promoted hepatocellular carcinoma cell invasion and migration in Huh7 cells compared with the pcDNA-control cells (*p* < 0.01) (Figure 3(a)). In HepG2 cells, knockdown of lncRNA SAMD12-AS1 (shSAMD12-AS1) significantly suppressed hepatocellular carcinoma cells' ability to migrate and invade compared with the shRNA-control cells (*p* < 0.01) (Figure 3(b)).

### 3.4. Effects of LncRNA SAMD12-AS1 Knockdown or Overexpression on the p53 Signaling Pathway in Hepatocellular Carcinoma Cells

**Figure 4:**
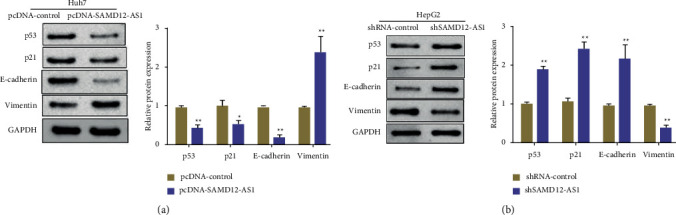
Effects of overexpression or knockdown of lncRNA SAMD12-AS1 on p53 signaling pathway in hepatocellular carcinoma cells: (a) overexpression of lncRNA SAMD12-AS1 inhibited the expression of p53, p21, and E-cadherin while promoted vimentin and (b) knockdown of lncRNA SAMD12-AS1 promoted the expression of p53, p21, and E-cadherin while inhibited vimentin. ^∗^*p* < 0.05, ^∗∗^*p* < 0.01.

The above results show that overexpression or knockdown of lncRNA SAMD12-AS1 has a significant effect on the invading and proliferating capability of liver cancer cells. Thus, we explored the molecular mechanism that regulates lncRNA SAMD12-AS1. First, Western blot was performed to evaluate the outcome of overexpression or knockdown of lncRNA SAMD12-AS1 on the expression of p53, vimentin, p21, and E-cadherin in liver cancer cells. It was found that lncRNA SAMD12-AS1 overexpression (pcDNA-SAMD12-AS1) inhibited the p53 and p21 of hepatocellular carcinoma cells and promoted cell proliferation in Huh7 cells compared with the pcDNA-control cells. Also, E-cadherin expression was suppressed, while expression of vimentin was promoted as well as the ability of cell invasion and migration (*p* < 0.01) (Figure 4(a)). In comparison with the shRNA-control group, lncRNA SAMD12-AS1 (shSAMD12-AS1) knockdown markedly inhibited the migratory and invasive ability of HepG2 cells (*p* < 0.01) (Figure 4(b)).

### 3.5. LncRNA SAMD12-AS1 Regulated the Proliferation and Migration of Hepatocellular Carcinoma via the p53 Pathway

**Figure 5:**
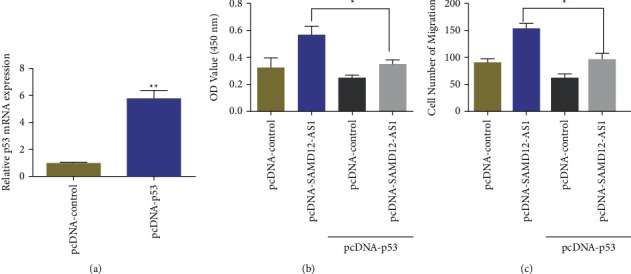
LncRNA SAMD12-AS1 regulated proliferation and migration of hepatocellular carcinoma via p53 pathway: (a) p53-overexpressed lentivirus was transfected in Huh7 cells and (b, c) overexpression of p53 altered the stimulating effect of lncRNA SAMD12-AS1 on the proliferation and migration of hepatocellular carcinoma cells. ^∗^*p* < 0.05, ^∗∗^*p* < 0.01.

To evaluate whether lncRNA SAMD12-AS1 played its function through p53 in hepatocellular carcinoma cells, rescue experiments of proliferation and migration were conducted. Real-time fluorescence quantitative PCR showed that transfection of the lentiviral vector (pcDNA-p53) significantly promoted the expression of intracellular p53 in Huh7 cells in comparison with the pcDNA-control cells (Figure 5(a)). In addition, CCK-8 data suggested that overexpression of lncRNA SAMD12-AS1 (pcDNA-SAMD12-AS1) significantly stimulated the migration and proliferation hepatocellular carcinoma cells, but overexpression of p53 significantly blocked the effect induced by overexpression of lncRNA SAMD12-AS1 (pcDNA-SAMD12-AS1) (Figures 5(b) and 5(c)). These data revealed that p53 can alter the promoting effect of lncRNA SAMD12-AS1 on the migration and proliferation of hepatocellular carcinoma cells.

## 4. Discussion

Existing studies have revealed that liver cancer is a malignant tumor with poor prognosis, and targeted therapy is a new method for the treatment of liver cancer at middle and advanced stages. It is important to determine more specific targets for clinical therapy against liver cancer. LncRNA has been discovered to be correlated with liver cancer development, metastasis, apoptosis, and incidence rate. This study found that the lncRNA SAMD12-AS1 expression was enhanced in liver cancer tissues compared with that in paracancerous liver tissue and was correlated significantly with tumor TNM stage, vascular invasion, and hepatitis B surface antigen in liver cancer patients. Cell function experiments show that lncRNA SAMD12-AS1 was significantly related to the liver cancer cell's proliferation, migration, and invasion. Molecular mechanism studies show that lncRNA SAMD12-AS1 modulates the liver cancer cell's development and their ability to invade by regulating the expressions of p21, p53, vimentin, and E-cadherin.

The characteristics of cancer cells are rapid proliferation and strong invasive and migratory ability. Thus, the invasion and migration capability is an important factor to judge the viability of cancer cells. For example, in tissues and cell lines of liver cancer, lncRNA TPT1-AS1 is highly expressed and is significantly linked to lymph node metastasis TNM stage and prognosis of patients. LncRNA TPT1-AS1 knockdown suppresses the growth, invading, and migrating ability of liver cancer cells [16]. By sponging miR-3118, lncRNA HAND2-AS1 enhances the JAK-STAT pathway, thereby promoting SOCS5 to inhibit the liver cancer cell's growth and migration [17]. The data from this investigation indicated that the level of lncRNA SAMD12-AS1 expression in liver cancer patients' tumor tissues was markedly increased than that in paracancerous liver tissue. Through analysis, we uncovered that the level of lncRNA SAMD12-AS1 expression was largely correlated with the TNM stage of the tumor, vascular invasion, and surface antigen of hepatitis B in patients with liver cancer. Cell function experiments showed that lncRNA SAMD12-AS1 overexpression could greatly enhance liver cancer cell proliferation, invasion, and migration, while lncRNA SAMD12-AS1 knockdown could inhibit the liver cancer cell proliferation, invasion, and migration.

Previous studies suggest that p53 is an important tumor suppressor protein and transcription factor in cells, which regulates cell division and prevent tumor formation. Some studies showed that p53 can directly affect gene transcription that regulates cell proliferation and migration. For example, p53 regulates transcription factors related to EMT such as Slug, Snail, and Twist, which can inhibit E-cadherin expression [18]. In addition, it was shown that some lncRNAs can play a tumor-promoting or tumor-suppressing function by regulating the expression of p53. For example, lncRNA PLAC2, by upregulating the expression levels of p53, can induce the development and promote the apoptosis of liver cancer cells [19]. The lncRNA HOXB-AS3 can stimulate hepatocellular carcinoma cell proliferation and inhibit apoptosis. It was revealed that the lncRNA HOXB-AS3 can bind to DNMT1 to suppress p53 expression and promote cancer progression [18]. The result of this study indicated that lncRNA SAMD12-AS1 can largely affect the p53 expression, that is, overexpression of lncRNA SAMD12-AS1 can markedly inhibit the p53 expression, while lncRNA SAMD12-AS1 knockdown can greatly enhance the p53 expression. The results of this investigation remain consistent with previous investigations, that is, lncRNA SAMD12-AS1 regulates the expression of p53 through different mechanisms and then exerts its regulatory function. For example, lncRNA SAMD12-AS1 in gastric cancer cells regulates the p53 expression through DNMT1, while in liver cancer cells through NPM1/HDM2 signaling axis [14,15]. However, the molecular mechanism of how lncRNA SAMD12-AS1 exerts physiological regulation through p53 is still unclear.

In addition, p21 is one of the important molecules in the transcriptional regulation of p53, and p21 is an important cell cycle regulatory protein responsible for proliferation and tumor cells [20]. Some drugs or genes can also inhibit tumor invasion and migration through p53. For example, in endometrial cancer cells, UBE2C regulates the expression of proteins associated with invasion and migration such as vimentin and E-cadherin through p53 [21]. Dulcitol suppresses liver cancer cells' ability to proliferate and migrate via MMP-2, uPA, MMP-9, and E-cadherin expression by regulating and modulating the SIRT1/p53 signaling pathway [22]. The results of this study showed that further research revealed that upregulation of lncRNA SAMD12-AS1 could markedly suppress the expression of p53, p21 and E-cadherin, promote vimentin expression, and enhance liver cancer cell proliferation and invasion. LncRNA SAMD12-AS1 knockdown can lead to increased expression of p53, p21, and E-cadherin and inhibit the expression of vimentin, thereby inhibiting the proliferation and invasion of hepatocellular carcinoma cells.

In conclusion, this study showed that lncRNA SAMD12-AS1 was highly expressed in liver cancer tissues and cell lines. In vitro cell function experiments demonstrated that overexpression or knockdown of lncRNA SAMD12-AS1 significantly affects the expression of p53, p21, E-cadherin, and vimentin, leading to altered cell proliferation, clone formation, invasion, and migration. This study confirmed that lncRNA SAMD12-AS1 plays an oncogenic role in liver cancer. Therefore, lncRNA SAMD12-AS1 could be a new gene target for precision treatment of liver cancer in the future.

## Figures and Tables

**Table 1:** Correlation between lncRNA SAMD12-AS1 expression and clinical characteristics of 32 liver cancer patients.

Characteristic	n	LncRNA SAMD12-AS1 expression	*P*
Gender			
Male	22	3.81 ± 1.13	0.5108
Female	10	3.54 ± 0.89	
Age, years			
<55	11	3.24 ± 0.75	0.2030
≥55	21	3.66 ± 0.92	
TNM stage (the 8th edition)			
I-II	19	3.04 ± 0.77	0.0490
III-IV	13	3.84 ± 1.43	
Tumor size (cm)			
<5	20	3.32 ± 0.87	0.2857
≥5	12	3.67 ± 0.95	
Tumor number			
1	24	3.47 ± 0.94	0.3893
>1	8	3.83 ± 1.21	
AFP (ng/mL)			
≤20	11	3.12 ± 0.76	0.0349
>20	21	3.90 ± 1.03	
Vascular invasion			
Yes	18	3.04 ± 0.83	0.0428
No	14	3.78 ± 1.15	
Tumor differentiation			
I-II	21	3.19 ± 0.88	0.1700
III-IV	11	3.68 ± 1.04	
HBs antigen			
Negative	10	2.98 ± 0.79	0.0434
Positive	22	3.81 ± 1.12	

Note. AFP, alpha-fetoprotein; the tumor differentiation is based on Edmondson–Steiner classification.

## Data Availability

E-mails could be sent to the following address to obtain the data: chengxiang@ntu.edu.cn.
